# Structures, Occurrences and Biosynthesis of 11,12,13-Tri-*nor*-Sesquiterpenes, an Intriguing Class of Bioactive Metabolites

**DOI:** 10.3390/plants11060769

**Published:** 2022-03-14

**Authors:** Víctor Coca-Ruíz, Ivonne Suárez, Josefina Aleu, Isidro G. Collado

**Affiliations:** Departamento de Química Orgánica, Facultad de Ciencias, Universidad de Cádiz, Puerto Real, 11510 Cádiz, Spain; victor.coca@uca.es (V.C.-R.); ivonne.suarez@uca.es (I.S.)

**Keywords:** 11,12,13-tri-*nor*-sesquiterpenes, isolation, biosynthesis, biological activity

## Abstract

The compounds 11,12,13-tri-*nor*-sesquiterpenes are degraded sesquiterpenoids which have lost the C_3_ unit of isopropyl or isopropenyl at C-7 of the sesquiterpene skeleton. The irregular C-backbone originates from the oxidative removal of a C_3_ side chain from the C_15_ sesquiterpene, which arises from farnesyl diphosphate (FDP). The C_12_-framework is generated, generally, in all families of sesquiterpenes by oxidative cleavage of the C_3_ substituent, with the simultaneous introduction of a double bond. This article reviews the isolation, biosynthesis and biological activity of this special class of sesquiterpenes, the 11,12,13-tri-*nor-*sesquiterpenes.

## 1. Introduction

The terpenoid family of natural products comprises thousands of compounds with high structural and stereochemical diversity deriving from a small number of linear isoprenoid precursors. Terpenes are built up from isopentenyl diphosphate, the universal precursor of all isoprenoids, and basic C_5_ isoprene units, which can be obtained either through mevalonate or 2-methylerythritol 4-phosphate pathways. Terpene structures are divisible into isoprene units (C_5_), which are linked in a head-to-tail manner [1]. They are classified into the following classes or groups based on the number of these isoprene units they contain: monoterpenoids, C_10_; sesquiterpenoids, C_15_; diterpenoids, C_20_; sesterterpenoids, C_25_, triperpenoids, C_30_; and carotenoids, C_40_ [2,3]. 

Among these, sesquiterpenes are the most numerous of the terpenoid compounds and can be grouped into approximately 30 major skeletal types, but at least 200 less common skeletal types are known. Sesquiterpene hydrocarbons are common essential oil components in plants and accumulate in many fungi species. In the sesquiterpene series, a-, mono-, bi-, tri- and tetra-cyclic compounds are known [4]. Of these, bicyclic and tricyclic predominate and they occur freely, although glycosides are also known in this series.

Cyclases transform 2-*E*-6-*E*-farnesyl diphosphate (FDP) into cyclic sesquiterpenes via ionization and electrophilic attack of the resultant allylic cation on either the central or distal double bond [2], yielding a wide variety of sesquiterpenic skeletons. The nature of the products eventually formed are a function of the stereochemistry and conformation of the intermediates, and the cyclases may serve as rate-controlling enzymes in sesquiterpene biosynthesis.

However, in the case of skeletons with two or more cycles, the immediate precursor is not FDP, but typically an intermediate formed from it (germacrene A/B) that undergoes initial protonation of the double bond. This causes the formation of a carbocation that triggers a cascade of reactions that explain the formation of skeletons, such as guaiane, eudesmane and, from the latter, the eremophilane skeleton.

These sesquiterpene skeletons can become degraded, losing the isopropenyl group situated at C-7. These compounds receive the name 11,12,13-tri-*nor*-sesquiterpenes, and some have exhibited interesting biological activities or played an important role in the environment or life cycle of different organisms.

In order to carry out the bibliographic search of this study, databases such as Scopus, Science Direct Elsevier, PubMed, Google Scholar and especially the CAS SciFinder^n^ platform were accessed to retrieve information, using several keywords: “sesquiterpene”, “natural sesquiterpenoids”, “tri-nor-sesquiterpene”, “trinor-sesquiterpene” and “tri-norsesquiterpene” to find all tri-nor-sesquiterpenes that were already known. We also included the words “biosynthesis” and “biological activity” in the search criteria to look for the information about the biosynthesis of the different families of tri-*nor*-sequiterpenes. From the search results, those compounds which presented a trinorsesquiterpene formula (C_12_H_n_O_n_) in the platform CAS SciFinder^n^ were indexed in this study, and articles that referenced that type of compounds were analyzed. Automatic search tools were used to exclude some of the articles, while others were screened manually. Papers published in languages other than English were excluded from the analysis, especially those written in Chinese and Japanese, except when there was an extensive summary of the article in English.

This review provides an overview of publication trends on structures, occurrences, isolation, biosynthesis and bioactivity of this degraded class of sesquiterpenes, i.e., the 11,12,13-tri-*nor*-sesquiterpenes. The information was retrieved up to February 2021 and 303 references were analyzed.

## 2. Tri-*nor*-Germacranes and Tri-*nor*-Elemanes

**Figure 1:**
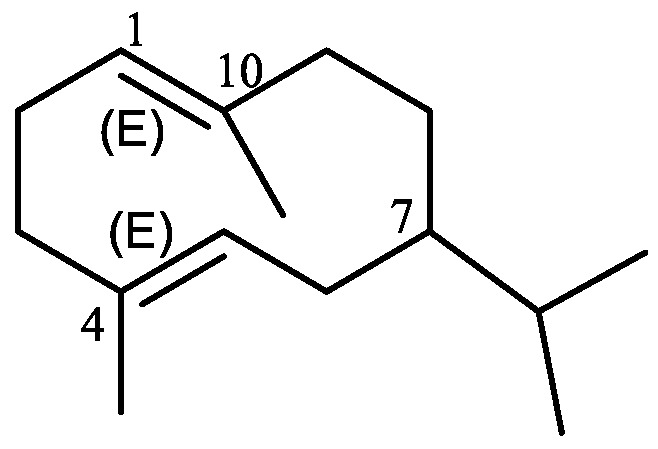
Molecular structure of 1(10),4-germacradiene.

Germacrane is the basic parent of a family of sesquiterpenes and is characterized by a cyclodecane ring structure substituted with an isopropyl group and two methyl groups. These sesquiterpenes are usually found in plant extracts as unsaturated derivatives with two double bonds at position 1(10) and 4, which are called 1(10),4-germacradienes (Figure 1). They are typically produced by a number of plant species and have antimicrobial and insecticidal properties [5].

Tri-*nor*-germacranes have the same skeleton as germacranes, except for the oxidative lack of the isopropyl group. Their properties are similar to those of germacranes, and this is why some tri-*nor*-germacranes can arouse commercial interest due to their biological properties.

**Figure 2:**
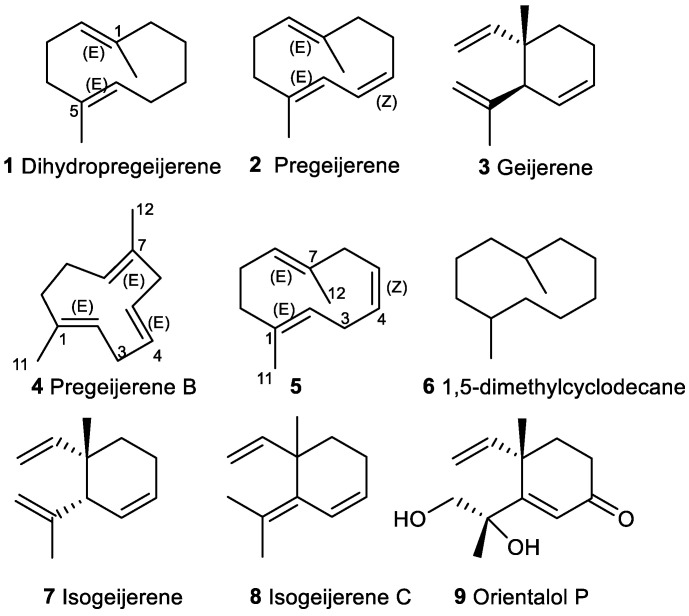
Molecular structure of 11,12,13-tri-*nor*-germacranes and -elemanes.

Many of the 11,12,13-tri-*nor*-sesquiterpenes identified are products of the secondary metabolism of many organisms. Most tri-*nor*-germacranes have been identified as components of essential oils (EOs) and some, such as compounds **1**, **2** and **4**–**6,** have been extracted from the essential oils of different plants (Figure 2).

Compound **1,** called dihydropregeijerene, is one of the tri-*nor*-germacranes that is a component of EO. Dihydropregeijerene (**1**) has been identified in the EO of *Fructus aurantii* [6]. A study about conformational isomerism in dihydropregeijerene (**1**) and hedycaryol has been reported (Figure 2) [7].

A re-examination of *Geijera parviflora* leaves, yielding geijerene (**3**) when worked up under standard conditions of steam distillation and fractional distillation, was found to contain a new hydrocarbon that was named pregeijerene (**2**) [8]. It was postulated to be a geijerene (**3**) precursor, as it conserved properties of the two compounds [8]. Hydrocarbon 2 formed a crystalline adduct with silver nitrate and rearranged thermally to yield geijerene (**3**).

Pregeijerene (**2**) has been isolated from the EO of different species of the Rutaceae family, in which *Ruta graveolens* is the most common plant and the one from which this compound has been studied [9,10,11,12,13,14,15,16,17,18,19,20,21,22,23,24].

It has also been extracted from *Rubus rosifolius* [25,26], a *Pimpinella* species [27,28,29,30,31,32,33,34,35,36,37,38,39,40,41,42,43,44,45,46,47,48,49,50,51,52,53], species of *Skimmia* [19,54,55,56,57,58,59,60], *Chloroxylon swietenia* [61] and other plant species.

Steam distillation of the leaves of *Boronia microphylla* provides an essential oil which contains pregeijerene (**2**) (Figure 2) [62]. This EO is full-bodied and fruity with a strong fragrance of *Vetiveria zizanioides* giving it a bitter, woody and grape-like odour. It is used as a componenet in Boronia perfume which has a fruity and woody note [62]. It has also been identified as a volatile fragrant component in a mini-core collection of mango germplasms from seven countries [63]. 

It has also been reported that compound **2** plays an important role in geosmine biosynthesis because, as mentioned above, pregeijerene (**2**) is an intermediate compound in geosmin biosynthesis [64].

Some essential oils containing pregeijerene (**2**), such as *Pimpinella khayamii* oil, exhibit interesting properties such as antimicrobial activity [49]. Oil samples from *Skimmia anquetilia* were tested for their biological properties and exhibited in vitro cytotoxic activity against four different cancer cell lines: viz MCF-7 (Breast), HeLa (cervix), PC-3 (Prostate) and Caco-2 (Colon), using a sulforhodamine (SRB) assay [58]. 

The antimicrobial and antioxidant activities of essential oils from *Pimpinella tragium* Vill. subsp. *glauca* (C. Presl.) (Apiaceae) have also been reported [52]. C-12 *nor*-sesquiterpenes were the principal class of metabolites (56.6–70.6%), among which pregeijerene (**2**) and geijerene (**3**) were predominant. Oil obtained from the stems exhibits the highest antibacterial activity, while oil from the flower is the most potent antioxidant [52].

A pregeijerene isomer known as pregeijerene B (**4**), (*E,E,E*)-1,7-dimethylcyclodeca-1,4,7-triene, has been identified in many different plant species. It was extracted for the first time from *Juniperus erectopatent* [65] and a common biosynthetic pathway for pregeijerene B (**4**), and the germacrene sesquiterpenoid 8-α-acetoxyhedycaryol was inferred from their co-occurrence in the foliage of 24 *Juniperus* species [65]. Similarly, in 2004, pregeijerene B (**4**) and 8-α-acetoxyelemol was proposed to arise from 8-α-acetoxyhedycaryol, accounting for their co-occurrence [66].

There was some resemblance of the Mass Spectrometry (MS) of compound **4** to that of the pregeijerene (**2**), but in contrast to the latter, which readily undergoes thermal rearrangement to geijerene (**3**) [8], pregeijerene B (**4**) remains stable even at 280 °C.

In addition to the isolation of compound **4** from the EO of *Juniperus* species [66,67,68,69,70,71,72], pregeijerene B (**4**) was also isolated from the EO of two endemic *Nepeta* species, namely *N. nuda* and *N. cadmea* [73]; the EO of *Helietta parvifolia*, which exhibited anticholinesterase activity [74]; and from different species of *Pimpinella* [43]. This compound was also isolated from the EO of *Stachys menthifolia* [75], *Artemisia annua* [76], *Calycanthus floridus* L. [77] and *Thottea ponmudiana* [78].

Regarding biological activity, oils from two *Juniperus* species have exhibited antifungal and insecticidal activity, and this bioactivity could be related to some of the properties of compound **4** as one of the components of the essential oil [71]. Some essential oils from *Juniperous, Nepeta* and *Artemisa* species exhibit antioxidant activity when compound **4** is one of the most abundant components [74,76,79,80,81]. 

Compound **4** has also been identified as the major component of oil extracted from the fresh leaves of *Thottea ponmudiana*, as well as *Nepeta ucrainica*, which was tested against both Gram-positive and Gram-negative bacteria. The oil showed significant activity against the Gram-positive bacteria *Staphylococcus aureus* and *Bacillus subtilis* in comparison to streptomycin [78,82]. Pregeijerene B (**4**) also appears in a patent for pharmaceutical compositions to treat chronic pain and opioid addiction [83].

Lastly, (*E,Z,E*)-1,7-dimethylcyclodeca-1,4,7-triene (**5**), isomer of pregeijerene B (**4**), was described as a dehydrogeosmin intermediate in its biosynthesis in *Cactaceae* flowers [64]. Some tri-*nor*-germacranes, i.e., compound 1,5-dimethylcyclodecane (**6**), were identified in the liposoluble constituents of *Paphia undulata* shell [84]. 

Geijerene (**3**) and isomers **7** and **8** are considered thermal artefacts of pregeijerene (**2**). Thus, it is known that pregeijerene (**2**) can be thermally isomerized to yield geijerene (**3**) by Cope rearrangement and chemical transformations (Figure 2). 

Compound **3** was extracted for the first time from the essential oils of some species of *Geijera* [85], and it was isolated in pure form from the linalool-geijerene azeotrope by an enhanced boratization procedure [86]. A structural study of geijerene, mainly by chemical degradation, led Sutherland to assign structure **3** for geijerene [87]. Its struture has also been studied independently by Birch et al., using an array of different physical methods [88]. Their confirmation that geijerene is correctly represented by **3** is especially valuable, since the occurrence of a plant product with two asymmetric centers in a racemic state is most unexpected. Further details of the degradations described in the earlier paper [87] and other confirmatory evidence, including a synthesis of the principal oxidation product of geijerene, have been analyzed in subsequent studies [89].

Owing to the many plants from which geijerene (**3**) has been isolated and the wide range of biological activities exhibited by the essential oils that contain this compound, this review only included the most significant examples. Geijerene (**3**) has been extracted from *Chloroxylon swietenia* DC leaves. The crude oil, whose principal compounds are germacrene D, pregeijerene (**2**) and geijerene (**3**), had a potent repellent effect on two mosquito species: *Aedes aegypti* and *Anopheles stephensi* [61,90,91,92,93]. Similar to pregeijerene (**2**), compound **3** has also been isolated from many *Pimpinella* species [42] and exhibits antimicrobial and antioxidant activity [52]. It has also been found in *Momordica charantia* [94] and in the essential oil of *Eupatorium odoratum* Linn. leaves and was found to be active against *E. coli* and *B. subtilis* [95]. Later, it was isolated from the essential oils of *Geijera parviflora* and *G. salicifolia*, where it exhibited antimicrobial and free radical scavenging activity [96]. It has also been isolated from the essential oils of two endemic *Nepeta* species, *N. nuda* subsp. *glandulifera* and *N. cadmea*. These essential oils have been shown to reduce metal ions and radicals. Moreover, both oils have relatively weak but noticeable activity against acetylcholinesterase and butyrylcholinesterase; they also have weak activity against α-glucosidase, but quite high activity against α-amylase and significant activity against tyrosinase [73]. Lastly, the chemical composition and antioxidant potential of essential oil from the seed kernel of *Moringa peregrine* were studied. Gas Chromatography (GC) and GC–Mass Spectrometry (MS) analyses of that essential oil revealed that it contains 33 compounds. Of these, geijerene (**3**) was identified as the major compound (33.38%). Study of its antioxidant activity indicated that *M. peregrine* essential oil can be considered as an alternative choice to synthetic antioxidants [97].

Compound **7**, known as isogeijerene, has only been detected in *Pimpinella* species [43] (Figure 2). The first evidence of the compound isogeijerene C (**8**) was from the chemical treatment of geijerene (**3**) with MeOH-KOH [87]. Birch et al. reported an isogeijerene prepared by the action of potassamide in liquid ammonia whose structure corresponded with isogeijerene C (**8**) [88]. 

Isogeijerene C (**8**) has been isolated from different species such as *Ruta graveolens* [10]. Interestingly, root callus and root organ cultures, whether grown in light or darkness, produced only geijerene (**3**) and pregeijerene (**2**), which are both present in intact roots, and isogeijerene C (**8**). Only dark stem callus cultures of *R. graveolens* predominately produced the terpenoid hydrocarbons geijerene (**3**) and pregeijerene (**2**) [11]. When these same cultures were changed from light to darkness or vice versa, the composition of the oils also changed, with isogeijerene C (**8**) being produced in the latter situation [11].

Some essential oils in which isogeijerene C (**8**) was detected exhibited anti-larval activity [98] and antioxidant, antimicrobial, anti-inflammatory and antifungal properties [95,99,100]. Isogeijerene C (**8**) has also been detected in the essential oil of *Pimpinella* species [41,42,43,45,50,101], *Agathosma* species [99], *Aspilia africana* [102], *Hymenocrater longiflorus* [98,100] and *Eupatorium odoratum* Linn [95].

We would note that there is a great deal of confusion in the literature concerning the names of compounds **3**, **7** and **8** found in different databases (Pubchem and Scifinder). Readers should, therefore, pay careful attention to references if interested in any of these compounds. 

Lastly, orientalol P (**9**) (Figure 2) was isolated from the rhizome of *Alisma orientale* (Sam.) Juzep [103]. The planar structure of **9** was determined to be 2,3-seco-11,12,13-tri-*nor*-eudesmane by extensive NMR spectroscopic methods. The relative stereostructure of this compound was correlated by NOESY experiment and named orientalol P.

## 3. Tri-*nor*-Eudesmanes: Geosmin and Derivatives

Many sesquiterpenes that lose the C_3_ unit at the C-7 position have an eudesmane skeleton. To help organize this discussion of the many tri-*nor*-derivatives isolated with an underlying eudesmane skeleton, in this section, we draw a distinction between derivatives which, themselves, have a eudesmane skeleton and geosmin derivatives.

### 3.1. Tri-nor-Eudesmanes: 11,12,13-Tri-nor-Eudesmanes

**Figure 3:**
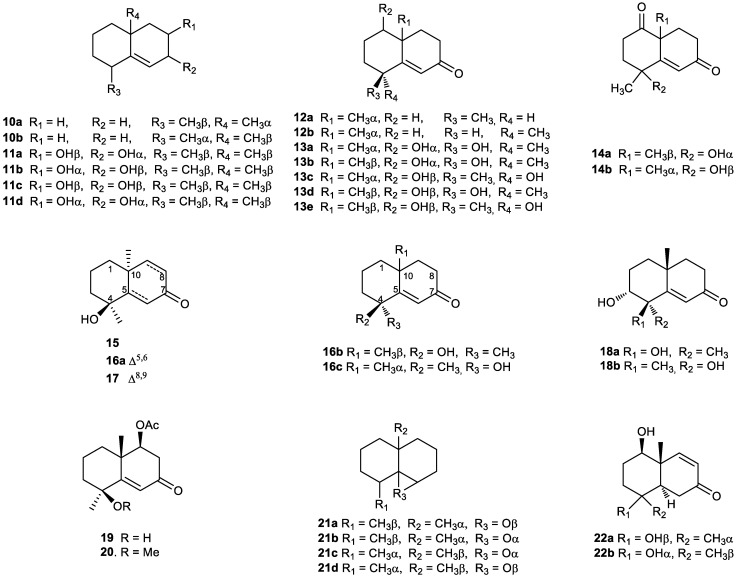
Several isolated and characterized 11,12,13-tri-*nor*-eudesmanes-type sesquiterpenes.

Interestingly, compound **10a**, which is an intermediate in the synthesis of geosmin [104], has subsequently been isolated from the liverworts *Lophocolea bidentata* and *L*. *heterophyla* [105] and from Taiwanese liverwort *Bazzania fauriana* [106]. Enantiomeric separation of synthetic **10a** and **10b** by preparative GC helped establish a correlation between configuration and optical rotation. GC investigations on a capillary column with the cyclodextrin derivative proved that the natural olefin **10a** was the (+)-enantiomer (Figure 3). Tri-*nor*-eudesmanes **11a**–**11c** were isolated from *Inula racemosa* [107,108,109]. Compound **11d** was isolated from the roots of *Inula helenium* [110]. The structures of isolated compounds were elucidated by extensive spectroscopic methods, including 1D and 2D NMR, and computational methods. Racemosin A (**11a**) was identified in *Inula racemosa* Hook. f [107], and it is an ingredient in several patented drugs to treat rhinitis [111], to treat or prevent myocardial ischemia [112], to treat epidemic haemorrhagic fever [113] and to treat or prevent acute heart failure (Figure 3) [114].

The diastereomers **12a** and **12b (**Figure 3) were isolated from the essential oils of *Vetiveria zizanioides* [115,116], and, therefore, they are components of Haitian vetiver oil [116]. Compound **12a** has been used as a reactant to achieve (−)-geosmin chemical synthesis [117,118,119,120,121]. It plays an important role in the cosmetic industry due to its scent [122].

Calamusin I (**13a**) was isolated from *Acorus calamus* rhizomes and exhibited weak hepatoprotective activity against APAP-induced HepG2 cell damage [123]. Tri-*nor*-eudesmanes **13b** and **13c** were isolated from the aqueous extract of *Alismatis Rhizoma* [124] and **13d** was isolated from *Teucrium polium* [125] and from *Alpinia oxyphylla* [126]. The structure of **13d** was identified by using standard MS and NMR spectroscopic methods. Its absolute stereochemistry was determined based on a modified Mosher’s reaction. The degraded sesquiterpene **13e** was isolated from the methanolic extract of the Red Sea soft coral *Litophyton arboreum*, along with known tri-*nor*-sesquiterpenoid teuhetenone A (**16a**) (Figure 3) [127]. Compounds **13e** and **16a** were assessed for their antimicrobial activity; both exhibiting weak activity against Gram-positive bacteria (*Bacillus subtilis* and *Staphylococcus aureus*). Furthermore, Gram-negative bacteria *Pseudomonas aeruginosa* and *Escherichia coli* were significantly inhibited by compounds **13e** and **16a** at minimum inhibitory concentration (MIC) values of 1.2 and 1.9 μg/mL, respectively. In particular, of the pure metabolites tested, only the *nor*-sesquiterpene **13e** was shown to exhibit moderate antifungal activity against *Candida albicans* with an MIC value of 3.2 μg/mL (Figure 3). Additionally, **13e** showed the most potent cytotoxic effect against MCF-7 cells with an IC_50_ value of 6.43 μM.

Compound **14a** was extracted from the aerial parts of *Teucrium ramosissimum* [128] and from the rhizomes of *Homalomena occulta* [129]. It exhibited significant in vitro antiplasmodial activity against *Plasmodium falciparum* with IC_50_ values of 3.3 μg/mL. However, no cytotoxicity was observed against the human diploid lung cell line MRC-5 for these compounds [128].

The compound named orientalol O (**14b**) was extracted from the rhizome of *Alisma orientale* (Sam.) Juzep [103]. Its structure and relative stereochemistry were elucidated by NMR spectroscopy (^1^H and ^13^C NMR, HSQC, HMBC and NOESY), electronic circular dichroism (ECD) and HR-ESI–MS data analyses. The nephrotoxicities of the isolated compounds were evaluated on normal human HK2 cells by high content screening, and neither the MeOH extract nor the compounds exhibited potential in vitro nephrotoxicity [103].

In a phytochemical study looking into species of the family Labiatae which are endemic to the Canary Islands, *Teucrium heterophyllum* L´Her was studied from a phytochemical point of view. The new 11,12,13-tri-*nor*-sesquiterpenes teuhetone (**15**), teuhetenone A (**16a**) and teuhetenone B (**17**) were isolated, and their structures were characterized by extensive mono- and bi-dimensional NMR techniques [130]. The tri-*nor*-eudesmanes **16a**–**16c** (Figure 3), were identified from *Alpinia oxyphylla* extract [131,132,133,134] and *Laggera alata* [135].

The 3,4-dihydroxy-α,β-unsaturated ketones oxyphyllenone A (**18a**) and B (**18b**) were isolated from the fruit of *Alpinia oxyphylla* (Figure 3) [136,137,138,139]. Compound **18a** had inhibitory effects on nitric oxide production; however, these compounds did not exhibit significant inhibitory activity against the release of β-hexosaminidase from RBL-2H3 cells [137]. 

Compounds **19** and **20** were extracted from liverwort *Apomarsupella revolute* [140], and their structures were established unequivocally on the basis of spectroscopic data analysis. The methoxy derivative **20** was considered an artifact of **19**.

Compound **21a** was isolated from the essential oils of mosses [141] and liverwort *Lophocolea bidentata* [105]. The structure and absolute configuration of **21a** was confirmed by synthesis from the olefin **10a**, obtaining the enantiomers **21c** and the couple **21b** and **21d** (Figure 3) [105].

The 1,4-dihydroxy-7-keto derivative **22a** was identified in *Alpinia oxyphylla* extract [126,134] and the rhizomes of *Homalomena occulta* [142] and *Teucrium ramosissimum* [128]. Structures and relative stereochemistry were elucidated by extensive spectroscopic studies, including 1D and 2D NMR and mass spectrometry (MS). Moreover, oxyphyllenone C (**22b**) was extracted from *Rhizoma cyperi* [143] (Figure 3).

The degraded eudesmane **23a** was obtained from the Tibetan folk medicine *Pulicaria insignis* [144,145]. This tri-*nor*-sesquiterpene exhibited weak inhibitory activity against the influenza virus H1N1 neuraminidase in an in vitro assay [146]. At a concentration of 200 mg/mL, compound **23a** showed 19.5 ± 1.4% inhibition. Unfortunately, **23a** proved to be very toxic against MDCK cells in the MTT assay. Further modification of the compound will be needed to reduce toxicity while increasing antiviral activity [144]. The structure of **23a** has been revised to structure **23e** [145], and the diastereomer **23b** was used as a precursor in the synthesis of cybullol (see geosmin derivative **34**) [147]. 

**Figure 4:**
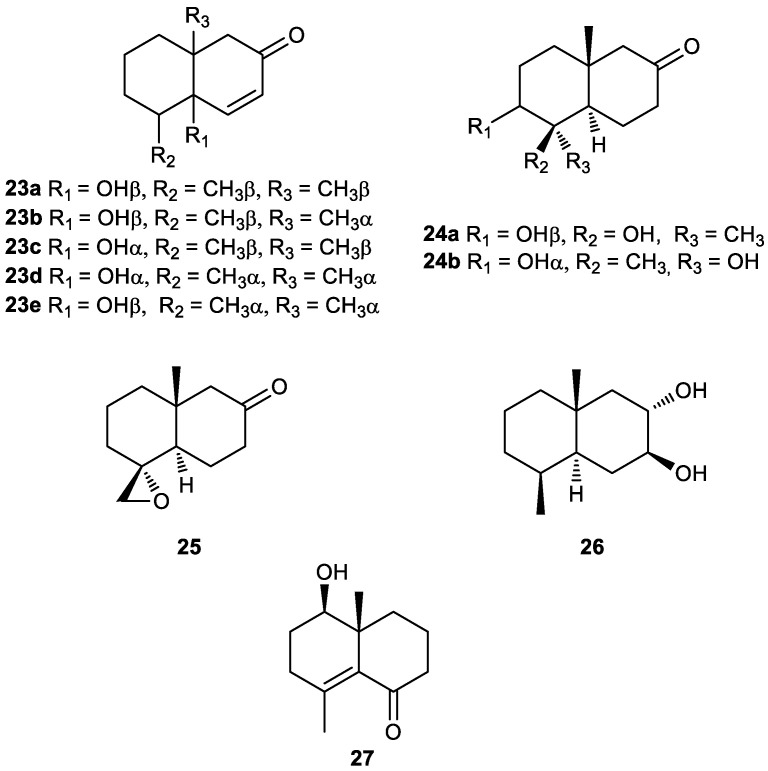
Some 11,12,13-tri-*nor*-eudemanes.

Compounds **23c** [109], **23d** and **23e** [145] were isolated from the roots of *Inula racemose* (Figure 4). The latter showed antiproliferative activity against A549, HepG2 and HT1080 cell lines with IC_50_ values of 3.71, 5.94 and 3.95 mg/mL, respectively [145].

The novel 11,12,13-tri-*nor*-3,4-diepicuauhtemone (**24a**) was isolated and characterized in a study of the fresh whole plant *Pluchea arguta* [148,149,150]. This compound, along with the diastereomer **24b**, has been described as an intermediate in the synthesis of cuauhtemone, a dihydroxy ketone sesquiterpene isolated from the Mexican medicinal shrub “Cuauhtematl” [151].

In addition to the tri-*nor*-sesquiterpenes **23c**–**23e**, compounds **25** and **26** were also isolated from the roots of *Inula racemosa* (Figure 4) [145]. All isolates were evaluated for their antiproliferative activities against three human cancer cell lines, using the CCK-8 cell viability assay. Unfortunately, compound **25** and **26** showed no such activity (IC_50_ > 50 mg/mL) against the tested cell lines.

Euphraticanoid D (**27**) (Figure 4) was isolated from *Populus euphratica* resins [152]. The structure of this new compound, including its absolute configuration, was characterized by spectroscopic, chemical and computational methods. Biological evaluation revealed that compound **27** exhibited neuroproctective activity in H_2_O_2_-induced HT-22 cells, with **27** occurring in a concentration-dependent manner.

Then the neuroprotective property of the isolate was assessed by using glutamate-induced SH-SY5Y cells, and it was found that compound **27** could dose-dependently provide protection from neural cell injury in a concentration range of 10–40 µM. A brief structure–activity relationship was briefly discussed [152].

### 3.2. Geosmin Derivatives

**Figure 5:**
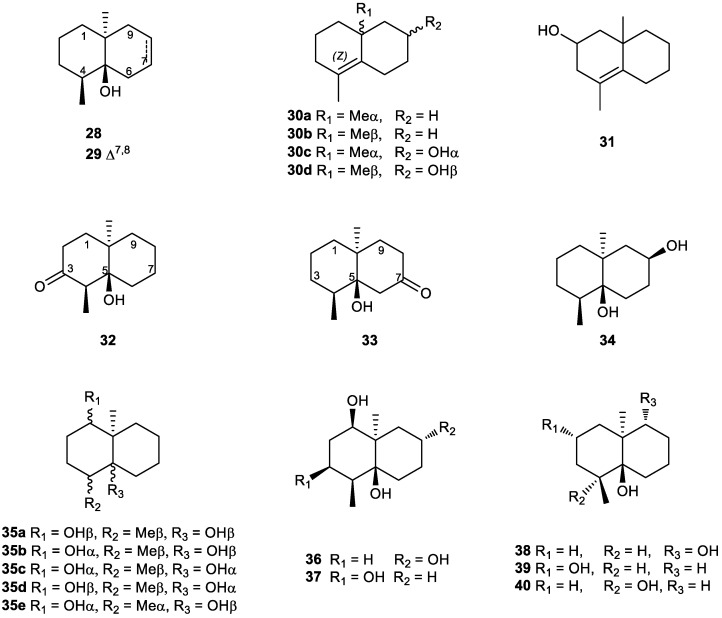
Geosmin and derivatives.

(−)Geosmin (**28**) (Figure 5) is a degraded sesquiterpene which has lost the isopropenyl group at seven position of the eudesmane skeleton, resulting in an 11,12,13-tri-*nor*-eudesmane. Its name comes from the Greek “ge”, meaning “earth”, and “osme”, meaning “odour” [153]. Geosmin was first isolated from the actinomycete *Streptomyces griseus* by Gerber and Lechevalier. This compound has a strong earthy smell with a low odour threshold of 10–100 parts per trillion that is produced by several microorganisms. It is responsible for the characteristic odour of freshly turned earth and is associated with unpleasant off-flavors in water [154,155,156,157], wine and fish [158]. 

It has also been found in fungi [159], including *Botrytis cinerea* and *Erysiphe necator* [160]. It is produced by different cyanobacteria [161,162,163,164] and myxobacteria, where geosmin (**28**) is responsible for the earthy smell of the culture [165]. Geosmin (**28**) has also been isolated from a variety of higher plants, such as liverwort and sugar beet [166], and from mosses, protozoans and insects [64,167].

It has been shown that, in contrast to flies, compound **28** does not repel mosquitoes (*Aedes aegypti*) but rather stimulates egg-laying site selection [168]. Environmentally relevant concentrations of geosmin (**28**) affect the development, oxidative stress, apoptosis and endocrine disruption of embryo–larval zebrafish [169]. 

(−)Geosmin (**28**) can be found at concentrations greatly exceeding its olfactory perception threshold in grape juices obtained from rotten grapes and in wine, indicating that it contributes to their earthy aroma [170]. 

In addition to compound **28**, several stereoisomers of (±)-geosmin have been described as intermediates in the synthesis of several natural products such as geosmin, dl-telekin and dl-alantolactone [171,172,173].

Dehydrogeosmin (**29**) (Figure 5) has been identified as the dominant olfactory compound in the scent of flowers of the Cactaceae species: *Rebutia marsoneri* Werd, *Dolichothele longimamma* (DC) Br et R., and *Sulcorebutia kruegeri* (Card) Ritt [174]. It has been identified as an aroma-active component of *Oenanthe javanica* and *Labisia pumila* essential oils [175,176]. It has also been identified in *Verbascum thapsus* [177]. Dehydrogeosmin (**29**) is an ingredient in pharmaceuticals, including tetrahydrocannabinol and cannabidiol for treatment of chronic pain and opioid addiction [83].

The sesquiterpenoid origin of dehydrogeosmin (**29**) has been reported based on the successful administration of deuterium-labeled farnesol to Cactaceae *Rebutia marsoneri* Werd and the metabolic conversion by flower heads of this plant [178].

Argosmin C (**30a**) has been obtained from different sources, but it was first detected by GC from the extract of the myxobacterium *Nannocystis exedens* [165]. Interestingly, it was obtained from an analysis of volatile organic biogenic substances (VOBSs) in freshwater phytoplankton populations [179] and algal blooms in South Australian waters [180]. This compound has also been detected in some moss species (Musci) [141] and identified by GC–MS from several sequenced actinomycetes (Figure 5) [120]. Its enantiomer **30b** was proposed as an intermediate compound in the photosensitized isomerizations of 10-methyl-1(9)-octalins [181]. Decaline **30c** has been described as an intermediate in the synthesis of artemisin [182]. It has been studied from the point of view of its structure–activity relationship, and it was found that minor structural changes had a major impact on odour. The enantiomer **30d** has been described as an important synthetic intermediate in alantolactone synthesis [171,172].

Compound **31** has been described as a chemical component in *Valeriana jatamansi* oil by GC–TOF-MS analysis [183].

Biotransformation of (±)-geosmin by the terpene-degrading bacteria *Pseudomonas* sp. SBR3-tpnd and *Rhodococcus wratislaviensis* DLC-cam yielded several products, with the major ones being (±)-3-ketogeosmin (**32**) and (±)-7-ketogeosmin (**33**) (Figure 5). Results suggest that the enzymes acting on geosmin enantiomers are not very site-specific and that compounds (±)-**32** and (±)-**33** are likely produced from (+)-geosmin [184]. Furthermore, geosmin’s derivatives, argosmin C (**30a**) and 3-ketogeosmin (**32**), were synthesized in an attempt to develop an ELISA for geosmin [185]. Results indicated that the binding of the antibody was restricted mainly to the bicyclic structure (A and B rings) of geosmin. The assay had a sensitivity of 1 µg/mL.

Cybullol (**34**), a C-8 hydroxyl derivative of geosmin, was isolated during the chemical study of the fungus *Cyathus buller*y Brodie, a species of gasteromycetous fungi known as bird’s nest fungi and widely distributed in nature (Figure 5). The structure was determined by a combination of chemical and physical methods. Its absolute configuration was deduced from the circular dichroism spectral of its ketol derivative and by chemical transformation to yield (−)-geosmin [186]. (±)-Cybullol (**34**) has been synthesized from 6,10-dimethyl-4-octal-3-one, and the transformation of 4,10-dimethyl-4-octal-3-one to (±)-geosmin was described by Ayer et al. [147].

The first total synthesis of 1β-hydroxygeosmin (**35a**) [187], a metabolite isolated from a fermentation broth of *Streptomyces albolongus* [188], was achieved via three different synthetic approaches from the racemic Wieland–Miescher ketone. The configuration of the hydroxyl groups at C-1 and C-5 was managed by using the Mitsunobu reaction and stereo- and regioselective epoxidation. Synthesis of stereoisomers **35b**–**35e** has also been described (Figure 5) [187]. Compound **35a** exhibited strong antifungal activity against *Candida parapsilosis* with a MIC value of 3.13 µg/mL. The odoriferous derivatives of geosmin **36** and **37** were also isolated from *S. albolongus* obtained from *Elephas maximus* feces [188]. Continuing with the quest for bioactive natural products from actinomycetes associated with animal feces, tri-*nor*-eudesmanes **38**–**40** (Figure 5) were isolated from *Streptomyces anulatus* derived from *Giraffa camelopardalis* feces [189]. The geosmin derivatives were not bioactive against four human cancer cell lines and did not have an inhibitory effect on lipopolyssacharide-induced NO production in RAW 264.7 macrophage cells.

## 4. Tri-*nor*-Eremophilanes: 11,12,13-Tri-*nor*-Eremophilanes

The family of eremophilane sesquiterpenes is widely distributed among different natural sources and has a wide range of biological activity, such as antitumor, anti-inflammatory and antimicrobial properties, among others. In recent years, new bioactive eremophilane sesquiterpenes have been discovered from various terrestrial and marine organisms [190].

Tri-*nor*-eremophilanes were first isolated from plants. The first known compound of this type was identified as a new C_12_-ketone, (+)-(1*S*, 10*R*)-1, 10-dimethylbicyclo [4.4.0]dec-6-en-3-one (**41**), isolated from Reunion vetiver oil from *Vetiveria zizanioides* (L.) Nash in 1972. The structure and absolute configuration of 41 were established by synthesis from (+)-isonootkatone [115]. 

**Figure 6:**
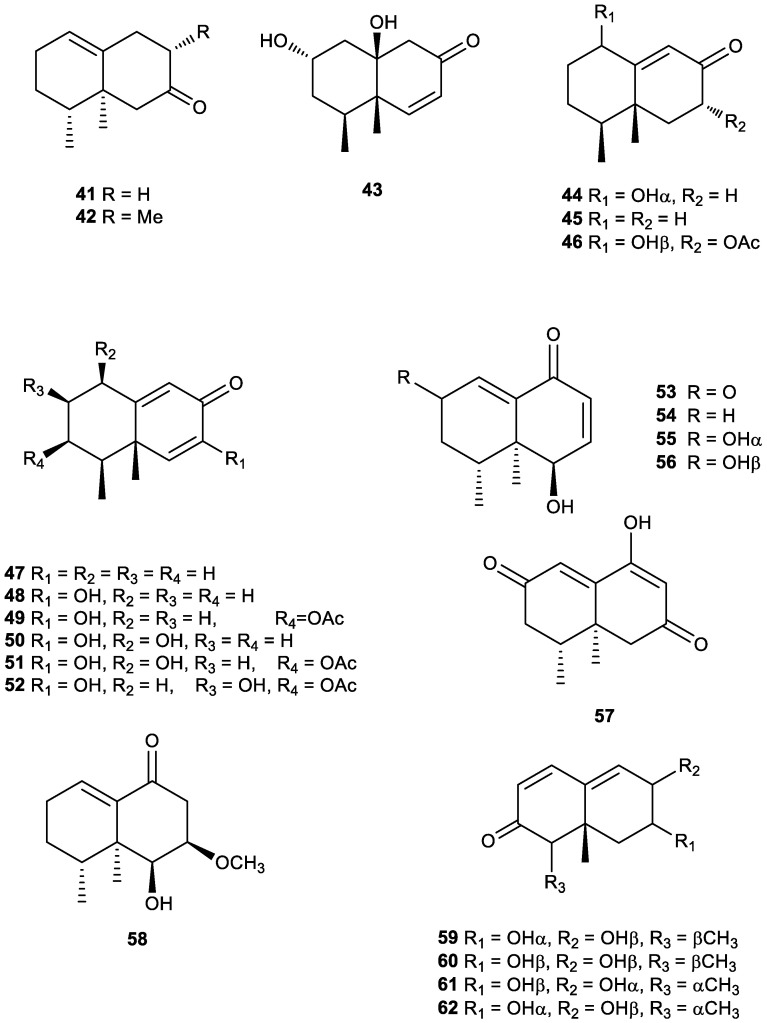
Some isolated 11,12,13-tri-*nor*-eremophilanes.

In 2000, Weyerstahl et al. described 155 components in the neutral part of commercial Haitian vetiver oil (*Vetiveria zizanioides*, Gramineae). Their structures were assigned mainly by ^1^H- and ^13^C-NMR spectra. The tri-*nor*-eremophilenone **41** was identified and named 11,12,13-tri-*nor*-eremophil-1(10)-en-7-one (**41**), and the new tri-*nor*-eremophilane, 8α-methyl-11,12,13-tri-*nor*-eremophil-1(10)-en-7-one (**42**) was also described (Figure 6). A sometimes unpleasant earthy off-note odour is typical for vetiver oil. The eremophilane derivative **42** revealed these unpleasant musty, earthy elements. In addition, **42** has a woody-camphoraceous odour [116].

The aerial parts extract of the South African plant *Ondetia linearis* was studied affording the two new tri-*nor*-sesquiterpenes 2α,10β-dihydroxyondetianone (**43**) and 1α-hydroxyisoondetianone (**44**), in addition to other known compounds. The structures were elucidated by high field NMR techniques. Compounds of this type are not common and are most likely the result of oxidative degradation, as this species appears to be very rich in oxidizing enzymes [191].

In 2009, Saito et al. reported for the first time the isolation of eremophilane-type compounds from the genus *Cremanthodium,* which is especially difficult to harvest, as it grows in high mountain areas. These authors were able to collect two samples of *Cremanthodium stenactinium* (Asteraceae) at different locations in Sichuan Province in China. The new tri-*nor*-eremophilane 4*S*, 5*R*-trinoreremophil-9-en-8-one (**45**) was isolated from the ethyl acetate extract of the roots (Figure 6). Its structure was determined based on spectroscopic data [192]. 

The genus *Ligularia* (Compositae) is widely distributed in China and has long been used in traditional folk medicine. This genus has antipyretic properties, loosens phlegm, relieves cough, invigorates blood circulation and sooths pain. Previous phytochemical studies on the genus *Ligularia* revealed that it is a rich source of eremophilane derivatives [193,194]. According to Chinese pharmacopoeia, *Ligularia* has been used to treat hemoptysis, rheumatism, pulmonary tuberculosis, urinary tract blockages, asthma, hepatitis and bronchitis for hundreds of years. Biological and phytochemical studies have shown that *Ligularia* species produce a variety of metabolites which have interesting structures and unique biological activities [195].

Two new tri-*nor-*eremophilane sesquiterpenes, (2*R*,5*R*,8*S*,8a*R*)-1,2,3,5,6,7,8,8a-octahydro-5-hydroxy-8,8a-dimethyl-3-oxonaphthalen-2-yl acetate (**46**) and (4a*S*,5*S*,8*R*)-5,6,7,8-tetrahydro-3,8-dihydroxy-4a,5-dimethylnaphthalen-2(4a*H*)-one (**50**), were isolated and identified as part of a study of the chemical components of the roots of *Ligularia sagitta* collected from the Gannan Tibet Autonomous Region in the Gansu Province of China (Figure 6) [196]. This compound **50** was also identified from the aerial parts of *Ligularia sagitta* [195].

Another similar derivative, tri-*nor*-sesquiterpene **47**, was isolated from the aerial parts of *Senecio humillimus* Sch. Bip. collected in Bolivia. Though its absolute configuration was not determined, the one proposed is very likely to be accurate as it is the one found in all of the eremophilane derivatives isolated thus far from members of the Compositae family [197].

The structure of a new *nor*-sesquiterpenoid was isolated from the roots of the perennial herb *Ligularia fischeri* collected in Nanchuan county of Chongqing city in China. The new compound was determined to be (4a*S*,5*S*)-5,6,7,8-tetrahydro-3-hydroxy-4a,5-dimethylnaphthalen-2(4a*H*)-one (**48**), a tri-*nor*-eremophilane sesquiterpene elucidated with the aid of key ^1^H, ^1^H-COSY and HMBC correlations [193]. 

The roots of *Ligularia przewalskii* have traditionally been used to relieve cough and asthma in Northwest China. Xu and Hu reported the study of this plant collected in Hefei City, Anhui Province, China, and the study resulted in the isolation of the new tri-*nor*-sesquiterpene 3β-(acetyloxy)-7-hydroxynoreremophila-6,9-dien-8-one (**49**) and three known eremophilane derivatives [194]. 

Bicyclic eremophilane-type sesquiterpenoids are mainly distributed in the *Ligularia* genus, but they are also present in other genera of the same Compositae family, such as *Senecio*. These natural products display multiple bioactivities, such as antisepsis, anti-inflammatory, anticancer and antineoplastic activity, and have also been used to treat cardiovascular disease. Not surprisingly, the synthesis of these compounds has attracted much attention among researchers. In 2018, Meng and Liu presented the successful syntheses of some natural products of this type, including compounds **48** and **50**. The syntheses feature a double Michael addition, Robinson annulation and α-enolization of an unsaturated ketone. The first total syntheses were achieved in three or four steps [198].

*Ligulariopsis* is a new genus Compositae represented only by *Ligulariopsis shichuana*, which is endemic to Western China. Previous studies of this plant have reported eremophilenolides and triterpenes, showing a close relationship between this species and those of *Cacalia* and *Ligularia* (Compositae). The acetone extracts of the whole dried plant of *L. shichuana* collected in Shaanxi Province, China, were separated to yield one new eremophilane with an 8-oxo-6,9-dien unit with no isopropyl group. This compound was established as 1β,7-dihydroxy-3β-acetoxynoreremophil-6(7),9(10)-dien-8-one (**51**) by spectroscopic methods and 2D NMR techniques [199].

Additionally, an isomer of compound **51** (Figure 6) was identified from the cultured endophytic fungus *Guignardia mangiferae*, which was isolated from the toxic plant *Gelsemium elegans* collected in Guangxi Province, China. This strain yielded the new tri-*nor*-sesquiterpene guignarderemophilane A (**52**). Its absolute configuration was determined on the basis of circular dichroism. This compound inhibited lipopolysaccharide-induced NO production in BV2 cells with an IC_50_ value of 15.2 μM (positive control curcumin, IC_50_ = 3.9 μM), showing anti-inflammatory activity [200].

Another genus with pharmacological relevance is *Nardostachys*. *Nardostachys jatamansi* (D.Don) DC. (family Caprifoliaceae, NJ) is commonly used in traditional medicine in China, India and Japan to cure digestive and mental disorders [201]. The rhizomes and roots of *Nardostachys chinensis* Batalin (Valerianaceae) have also been used as a sedative and analgesic in traditional Korean medicine. Modern pharmacological studies have shown that natural products from this plant exhibit bioactivity against depression, arrhythmia, convulsion, myocardial ischemia and hypertension [202,203].

An analysis of the methanolic extract of roots and rhizomes of *Nardostachys chinensis* Batalin led to the isolation of the new tri-*nor*-sesquiterpenic diketo-alcohol narchinol A (**53**), whose stereostructure was deduced on the basis of chemical and physical data [204]. Subsequently, desoxonarchinol A (**54**) was isolated for the first time from the same species and exhibited cytotoxic activity against P-388 cells [205].

In the search for new inhibitors of nitric oxide (NO) production from plants, Hwang et al. found that a methanolic extract of *N. chinensis* potently inhibited NO production in LPS-stimulated RAW 264.7 cells, indicating anti-inflammatory activity. Bioassay-guided fractionation of the CH_2_Cl_2_-soluble fraction of *N. chinensis* led to the isolation of two new sesquiterpenoids, namely narchinol B (**55**) and narchinol C (**56**) (Figure 6), along with other known compounds [202]. 

The compounds desoxonarchinol A (**54**) and narchinol B (**55**) also inhibited excessive production of proinflammatory mediators and pro-inflammatory cytokines in LPS-stimulated BV2 and primary microglial cells, proving that they are potential candidates for the development of therapeutically relevant agents to prevent neurodegenerative disease [206]. Additionally, compounds **53** and **55** had a protective effect on neonatal rat cardiomyocyte injury induced by hydrogen peroxide [207].

*Nardostachys jatamansi* contains several types of sesquiterpenes with potential anti-inflammatory activity. Thus, Yoon et al. studied the methanolic extracts of this plant and isolated the new nardosinone-type compounds kanshone M (**57**) and 7-methoxydesoxonarchinol (**58**), along with the known narchinol A (**53**) [208]. Compounds desoxonarchinol A (**54**) and narchinol B (**55**) were also isolated from the roots and rhizomes of this species [209]. 

Chaetopenoid F (**59**) was identified in the endophytic fungus *Periconia* sp. F-31, which was originally isolated from the medicinal plant *Annona muricata*. Three stereoisomeric tri-*nor*-eremophilane sesquiterpenes, periconianones I−K (**60**–**62**) (Figure 6), were also isolated from the same strain. These structures, including absolute configurations, were elucidated through extensive spectroscopic data analysis and electronic circular dichroism. Compound **62** exhibited anti-inflammatory activity indirectly by suppressing LPS-induced NO production in BV2 cells with inhibition rates comparable to those of curcumin, the positive control. Compound **59** exhibited low cytotoxic activity against the HeLa cancer cell line, and low anti-HIV activity with an IC_50_ value of 11.0 μM, whereas the positive control efavirenz had an IC_50_ of 1.4 nM [190]. 

**Figure 7:**
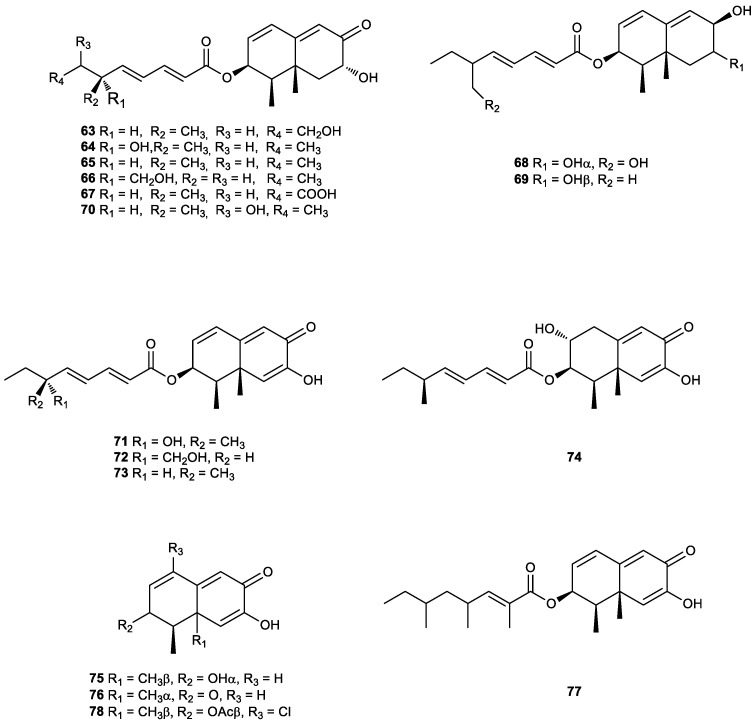
Molecular structure of 11,12,13-tri-*nor*-eremophilane derivatives.

As seen so far in this review, truncated eremophilanes lacking the isopropyl group have mostly been isolated from terrestrial plants, but in 1988, study of the secondary metabolism of the marine deuteromycete *Dendryphiella salina* strain led to the isolation and characterization of the first tri-*nor*-eremophilane, dendryphiellin A (**63**), esterified by a branched C_9_ acid, a class of metabolite for which there is no precedent in fungi of marine origin (Figure 7) [210].

In subsequent work, the same researchers reported the isolation of novel tri-*nor*-eremophilanes called dendryphiellin B (**64**), C (**65**) and D (**66**) (Figure 7) with spectral features that closely resemble those of dendryphiellin A [211]. In addition, dendryphiellin A1 (**67**) was subsequently isolated from the same *D. salina* strain [212]. 

Dendryphiellin A1 (**67**) was also identified in the culture broth of the Hawaiian endophytic fungus *Chaetoconis* sp. FT087 that was isolated from the leaves of *Osmoxylon novoguineensis* (Scheff.) Becc. This compound exhibited moderate antiproliferative activity against A2780 and cisplatin resistant A2780CisR cell lines, with IC_50_ values of 6.6 and 9.1 µg/mL, respectively [213]. Moreover, two other new tri-*nor-*eremophilanes were isolated from this endophytic fungus, namely chaetopenoids D (**68**) and F (**59**) (Figure 6 and Figure 7), but none of them exhibited either anti-proliferative or antibacterial activity [213].

The plant pathogenic fungus *Septoria rudbeckiae* Ellis and Halst (Mycosphaerellaceae) was isolated from the halophyte *Karelinia caspia*, a perennial shrub collected in the Xinjing Uyghur Autonomous Region of Western China. The study of this strain afforded 11 eremophilane sesquiterpenoids with a tri-*nor*-eremophilane skeleton: four known compounds, dendryphiellin B (**64**), C (**65**) and D (**66**) (Figure 7); and chaetopenoid F (**59**) (Figure 6), and seven new ones called septeremophilanes B–H (**69**–**75**). Their structures and absolute configurations were established based on spectroscopic data (NMR and HRESIMS), quantum chemical calculations and electronic circular dichroism (ECD) experiments. All metabolites were tested for nitric oxide (NO) production inhibition in lipopolysaccharide (LPS)-activated BV-2 microglial cells, and dendryphiellin D (**66**), septeremophilane D (**71**) and septeremophilane E (**72**) were found to display significant inhibition. These results contribute to the development of more effective drugs to treat neuroinflammation [214].

Other compounds with similar structures and the same backbone have been isolated from other sources. Thus, the trinorsesquiterpenic diketo-alcohol botryosphaeridione (**76**) (Figure 7) was identified for the first time from the endophytic fungus *Botryosphaeria rhodina* PSU-M35, which was isolated from the leaves of *Garcinia mangostana* collected in Suratthani Province, Thailand [215], while compound 76 was isolated from *Phoma* sp. LN-16, an endophytic fungus associated with *Melia azedarach*, growing on the campus of Northwest A&F University, Yangling, Shaanxi province, China. The first unequivocal assignment of its absolute configuration, (−)-(5*R*, 6*S*)-**76,** was made by circular dichroism spectra and was also established by means of X-ray diffraction. Moreover, that was the first report of a tri-*nor*-eremophilane sesquiterpene isolated from the *Phoma* genus.

This compound exhibited a strong inhibiting effect on lettuce seed germination (*Lactuca sativa*) [216].

The study of the phytopathogenic fungus *Lasiodiplodia theobromae* that was isolated from infected guava in Brazil resulted in the identification of the new tri-*nor*-eremophilane-type sesquiterpene **77**. This is the first time that an eremophilane sesquiterpene was described for the *Lasiodiplodia* genus [217].

A new chloro-tri-*nor*-eremophilane sesquiterpene (**78**) (Figure 7) was obtained from a fungus identified as *Penicillium* sp. PR19N-1 from deep-sea sediment collected in Antarctica. This is the first example of this kind of compound associated with microorganisms in the past 30 years. This novel tri-*nor*-eremophilane exhibited moderate cytotoxic activity against human leukemia HL-60 and lung cancer A-549 cell lines. These results show that, in the case of deep-sea fungi inhabiting the Antarctic, the extreme conditions lead to the expression of unusual biosynthetic mechanisms that could lead to unique secondary metabolites. Undeniably, the exploitation of these peculiar metabolic pathways represents a new opportunity for the discovery of bioactive secondary metabolites [218].

## 5. Tri-*nor*-Guaianes: 11,12,13-Tri-*nor*-Guaianes

**Figure 8:**
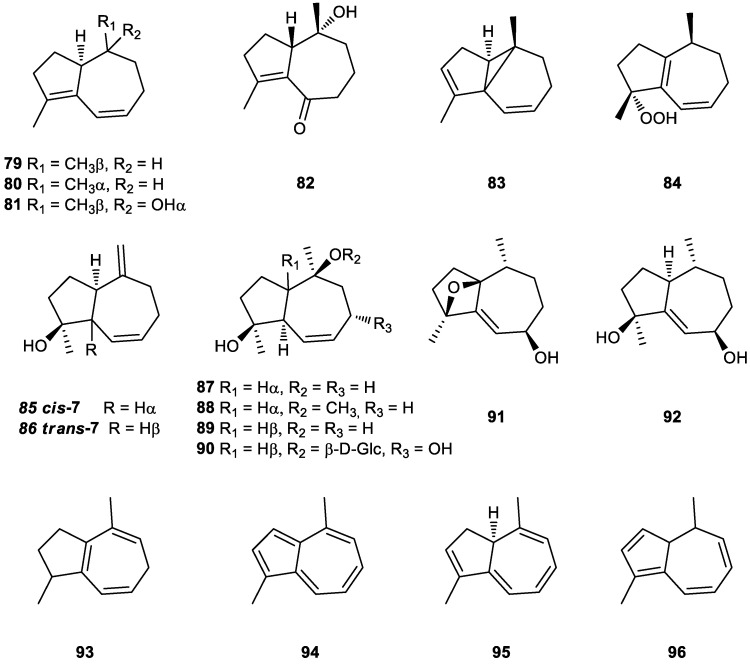
Molecular structure of 11,12,13-tri-*nor*-guaianes isolated and characterized.

Natural tri-*nor*-guaianes are rare metabolites that have been isolated from both terrestrial and marine sources. One of their most representative members is (−)-clavukerin A (**79**) (Figure 8), an unstable diene isolated from the Okinawan soft coral *Clavularia koellikeri* by Kobayashi et al. [219] during a search for biologically active compounds from marine sources. Its absolute stereochemistry was determined by spectral methods and by X-ray analysis of its diepoxide [219]. 

Bowden et al. reported the isolation of a terpenoid from an Australian soft coral *Cespitularia* sp. [220], which was later identified as **79** [221].

The first total synthesis of (−)-clavukerin A (**79**) was reported by Asaoka in 1991 [221], and it was then followed by several other racemic [222,223,224,225,226] and enantioselective syntheses [227,228,229,230,231,232,233,234].

Subsequently, in 1992, Kusumi et al. reported the isolation and structure elucidation of isoclavukerin A (**80**), an epimer of **79**, from the Okinawan soft coral *Clavularia* species. Its absolute configuration was established by a combination of CD and modified Mosher’s methods [235].

Several total syntheses of isoclavukerin A (**80**) have been reported (Figure 8) [221,223,224,232,233,236], confirming its structure. Hydroazulenes **79** and **80** have often been used as a testing ground for novel synthetic methods and strategies [221,222,223,224,225,227,229,230,231,232,233,236,237].

The tri-*nor-*guaiane (−)-2,3,3a,4,5,6-hexahydro-1,4-dimethylazulen-4-ol (**81**), a hydroxylated derivative of clavukerin A (**79**), was first isolated as a trace component of the essential oil of the liverwort *Barbilophozia floerkei* collected from the Harz mountains near Altenau, Germany [238].

Recently, Liu et al. studied the resins secreted by the tree *Populus euphratica*, which have been used to treat tuberculous adenitis, throat and duodenal ulcer swelling in China. In that work, a new tri-*nor*-guaiane, euphraticanoid C (**82**), was isolated and characterized by spectroscopic, chemical, and computational methods. The neuroprotective properties of this compound were observed in glutamate-induced SH-SY5Y cells and proved that euphraticanoid C (**82**) could dose-dependently protect neural cell injury [152].

Trinoranastreptene (**83**), which was first isolated from the cultured cells of the liverwort *Calypogeia granulata* Inoue (Figure 8) [239], is a tricyclic tri-*nor*-sesquiterpene that has an unprecedented tricyclo[5.3.0^1,6^.0]decane ring system. Its structure was determined by detailed NMR analysis, and it turned out to be identical or antipodal to the clavukerin B from Okinawan soft coral (stolonifer) *Clavularia koellikeri* [240,241] and inflatene from the stoloniferan coral *Clavularia inflata* var. *Luzoniana* collected in Palau, Western Caroline Islands, which exhibits ichthyotoxicity toward the Pacific damselfish *Pomacentrus coeruleus* [242]. To confirm its structural assignment, Kang et al. [243] performed a total synthesis of racemic trinoranastreptene (**83**), a surprising and interesting carbon skeleton.

Essential oils of the genus *Pimpinella*, a plant genus represented by approximately 150 species distributed throughout Europe, Asia and Africa, are complex mixtures that contain sesquiterpenes, phenolic compounds and alkenes [52]. In characterizing several *Pimpinella* species based on the qualitative and quantitative chemical patterns of their extracts, Kubeczka et al. studied the essential root oil of *Pimpinella* *major* [34] and *Pimpinella* *saxifrage* L. [30]. Moreover, Velasco-Negueruela et al. used gas chromatography–mass spectrometry to characterize the essential oils from the aerial parts of *Pimpinella anagodendron* Bolle and *Pimpinella rupicola* Svent., two species endemic to the Canary Islands, Spain [39]. Trinoranastreptene (**83**) was found in all the extracts.

Similarly, extracts from *Pimpinella* species collected from Turkey [41,43,50] were analyzed, and trinoranastreptene (**83**) was identified, along with more than 140 other different compounds.

Subsequently, Maggio et al. reported on the chemical composition and antioxidant and antimicrobial activities of the hydrodistilled essential oils from the flowers, leaves and stems of *Pimpinella tragium* Vill. subsp. *glauca* collected from Sicily (Italy). Trinoranastreptene (**83**) was found mostly in the flower extract and proved to be the most potent antioxidant [52].

Many research groups have studied liverworts from the Lophoziaceae family, as they are a rich source of terpenoids. Thus, tri-*nor*-guaiane **83** was identified in the ether extract of *Lophozia ventricosa* [244,245,246] and of *Barbilophozia floerkei* [238]. It has also been identified in tobacco smoke [247].

Clavukerin C (**84**) (Figure 8), an interesting tri-*nor*-guaiane with a hydroperoxy function, was extracted for the first time from *C. koellikeri* [240,241]. The presence of the hydroperoxyl function was suggested by the positive reactions with *N,N*-dimethyl-*p*-phenylenediammonium dichloride reagent and ferrous thiocyanate reagent [241]. It is also an intermediate of the synthesis of clavukerin A (**79**) [227]. Clavukerin C (**84**) was obtained from clavukerin A by photo-oxidation [222]. 

Moreover, a new tri-*nor*-guaiane type sesquiterpene named dictamnol, an active ingredient in Chinese medicines used for the treatment of various diseases, was first isolated from the roots of *Dictamnus dasycarpus* Turcz [248]. These authors later confirmed the structure of **85** by total synthesis [249].

However, De Groot et al. later performed a total synthesis of *cis*-dictamnol (**85**) and, owing to differences in the spectroscopic data of the synthetic compound and natural dictamnol, these authors proposed a revised structure for the natural product with a *trans*- (**86**) and not a *cis*-fused hydroazulene system (**85**) [250].

Dictamnol (**86**) features a core ring system common to a wide range of interesting natural and synthetic compounds. Thus, Wender et al. described its asymmetric synthesis based on a cycloaddition methodology in order to define the limitations and utility of these kinds of reactions [251].

Since then, compound **86** has been extracted from several *Pimpinella* species [42,43,45,47,52,252,253,254] and *Dictamnus* species [255,256,257]. 

Essential oil from the shoots of *Kochia scoparia* (L.) Schrad has traditionally been used in Chinese medicine to treat skin diseases, diabetes mellitus and rheumatoidal arthritis in Korea. El-Shamy et al. analyzed the volatile oil, which had a broad antibacterial spectrum and moderate antifungal activity. Dictamnol (**86**) was identified in the extract as a major component [258]. This compound was also found in the essential oil of several *Agathosma* species indigenous to South Africa that exhibited antimicrobial, anti-inflammatory and cytotoxic activities [99].

In 2005, Xiang et al. isolated a new tri-*nor*-guaienediol from the aerial parts of the plant *Siegesbeckia orientalis* L. used in traditional Chinese medicine to treat malaria, rheumatic arthritis, hypertension and other diseases [259]. Subsequently, Zhao et al. found the same compound in the extract of *Dictamnus radicis* root and named it radicol (**87**) [256]. It was also identified as a chemical component of the medicinal species *Dictamnus dasycarpus* [260] and *Dictamnus angustifolius* [257]. 

Similarly, compound **87** was identified in extracts from the aerial parts of *Pimpinella tragium* collected from Turkey [253] and was also found for the first time among the chemical components of the invasive plant *Chromolaena odorata* (L.) [261].

Recently, Li et al. determined that radicol (**87**) was highly cytotoxic to temozolomide-resistant glioblastoma multiforme cell lines and identified the potentially pro-apoptotic mechanism. These authors considered radicol (**87**) as a promising agent for the treatment of malignant gliomas because of its cytotoxicity to multiple targets, low molecular weight and high lipid solubility [262].

The radicol methoxy derivative, kanalpin (**88**) (Figure 8), was isolated from the methanolic extract of *Pimpinella cappadocica*. Its antioxidant capacity was evaluated, and kanalpin (**88**) was found to be inactive [263].

The *trans*-radicol, the tri-*nor*-guaiane 4β,10α-dimethyl-1β,5α−bicycle[3,5,0]dec-6-en-4α,10β-diol (**89**), was isolated for the first time from *Ainsliaea fragrans* Champ. [264] and Ding et al. later confirmed its structure by single crystal X-ray diffraction, identifying it in extracts from the leaves of *Magnolia grandiflora* [265].

Previously, in 2001, a tri-*nor*-guaiane-type sesquiterpene glycoside, dictamnoside N (**90**), was isolated from the water-soluble components of the root bark of *Dictamnus dasycarpus* [266], a traditional Chinese medicine used to treat jaundice, cough, rheumatism and some skin diseases. Sugar moiety was determined as β-D-glucose by acid hydrolysis and comparison with an authentic sample.

In subsequent studies, the structures and absolute configurations of two new trinorguaiane sesquiterpenes, claruviridins A (**91**) and B (**92**) (Figure 8), were determined by means of X-ray diffraction analysis. These metabolites were isolated from the Xisha soft coral *Clavularia viridis*, which can be found in the waters of the South China Sea [267]. Claruviridin B (**92**) was evaluated for its antitumoral activity and was found to be mildly cytotoxic against A549 cell lines.

In 2015, Hanif et al. reported on a “new” compound with the same structure as claruviridin B (**92**) [268]. However, an overall comparison of the NMR data of the two compounds unexpectedly showed that the structures were different, indicating that the metabolite isolated by Hanif was a stereoisomer of compound **92** [267]. This metabolite, whose stereochemistry has yet to be elucidated, was mildly cytotoxic against NBT-T2 rat bladder epithelial cells [268].

Furthermore, 1,4-dimethylazulenes has the same structure as tri-*nor*-guaian sesquiterpenes. Compound (+)-1,2,3,6-tetrahydro-1,4-dimethylazulene (**93**) was isolated for the first time from the essential oil of the liverwort *Barbilophozia floerkei* collected from the Harz Mountains near Altenau, Germany [238].

In 1966, Meuche et al. isolated the compound identified as 1,4-dimethylazulene (**94**) from the lichen *Calypogeia trichomanis*. Its structure was confirmed by synthesis [269]. 

Subsequently, this metabolite 94 was identified, together with other compounds, in many extracts and essential oils. Thus, 1,4-dimethylazulene (**94**) was produced as the major volatile metabolite in the cultured cells of *Calypogeia granulata* Inoue, a leafy liverwort [239,270]. This novel azulenoid compound had also been obtained from the aerial parts of *Helychrisum acuminatum* [271] and from the essential oil of the liverwort *Barbilophozia floerkei* collected in Germany [238]. Compound **94** has also been extracted from the essential root oil of *Pimpinella* species [30,34,41,42,43,50], and it has also been identified in cannabis smoke [247].

Furthermore, 3,10-Dihydro-1,4-dimethylazulene (**95**), a labile tri-*nor*-sesquiterpene biosynthetic precursor of 1,4-dimethylazulene (**94**), was first isolated from a cell culture of the liverwort *Calypogeia granulata* [239,272]. Its absolute stereochemistry was determined by the theoretical calculation of its circular dichroism spectra and verified by the synthesis of model compounds [273]. 

Compound **95** has also been identified in extracts from *Pimpinella* [30,41,43,50], in *Eupatorium odoratum* species [274] and in the oil of Moroccan chamomile *Cladanthus mixtus* (L.) Chevall [275].

An isomer of 95, compound 4,10-dihydro-1,4-dimethylazulene (**96**) (Figure 8) was identified by analysis of essential oils from several *Pimpinella* species [41,42,43,50].

## 6. Miscellaneous Tri-*nor*-Sesquiterpenes

Here we briefly discuss the tri-*nor*-sesquiterpenes that cannot easily be assigned to a particular structure class with the typical skeleton of the four families of sesquiterpenes previously reported: germacranes, eremophilanes, eudesmanes and guaianes. These types of tri-*nor*-sesquiterpenes are synthesized by numerous organisms, and some exhibit pharmaceutical properties attracting commercial interest. However, our knowledge of them is limited, and some of their properties are still unknown. 

**Figure 9:**
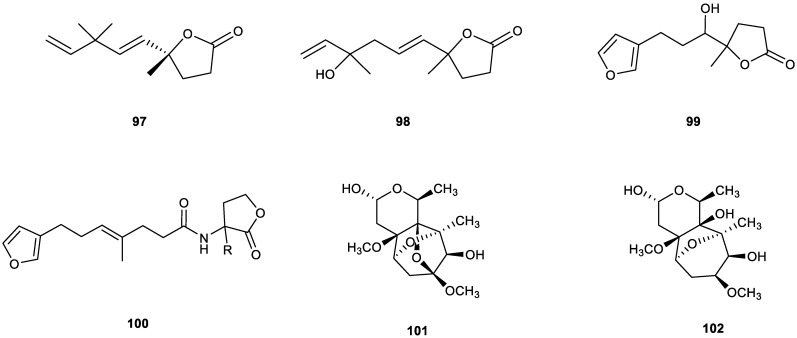
Other 11,12,13-tri-*nor*-sesquiterpenes.

Having studied the constituents of a plant from Costa Rica, *Calea prunifolia* H.B.K., Castro et al. reported the isolation of a complex mixture of hydrocarbons. The aerial parts afforded the tri-*nor*-sesquiterpene lactone apocalepruna-1,4*E*-dien-6,9-olide (**97**) (Figure 9), a derivative of a hitherto unknown sesquiterpene type. The structure was elucidated by spectroscopic methods [276].

Later, another tri-*nor*-sesquiterpene lactone, crocinervolide (**98**), was first isolated from the aerial parts of *Calea crocinervosa* when the plant was in bloom [277]. It has also been extracted from two *Gonospermum* species, *G. gomerae* and *G. fruticosum*, together with other known compounds [278], and from of the aerial parts of *L. sinense* cv. *Chaxiong* [279]. This compound was also isolated from the endophytic fungus *Umbelopsis dimorpha* SWUKD3.1410 and from its host-plant *Kadsura angustifolia* [280]. Crocinervolide (**98**) (Figure 9) has also been reported as a component of polymers and prepolymers used for contact lenses. Natural compounds are used in contact-lens polymers to reduce eye injury, inflammation and allergic reactions associated with long-term use [281].

Although furanoterpenoids are a class of frequently encountered natural products in marine invertebrates, this type of metabolite containing butanolide motif was rarely reported. In particular, tri-*nor*-sesquiterpenoids bearing both furan and butanolide moieties are unprecedented. Two rare new furan butanolides, sponalisolides A (**99**) and B (**100**) (Figure 9), were isolated in racemic forms from the marine sponge *Spongia officinalis* and are the first examples of such terpenoids found in Nature. Their structure, including the absolute stereochemistry of the two pairs of enantiomers, were unambiguously established by biomimetic total synthesis, involving a key Johnson–Claisen rearrangement and a lactone cyclization. All the sponalisolide enantiomers exhibited *Pseudomonas aeruginosa* quorum-sensing inhibitory activity [282].

Two tri-*nor*-sesquiterpenoids, urechitols A (**101**) and B (102), were isolated from the methanolic root extract of *Pentalinon andrieuxii*, a plant commonly used in Yucatecan traditional medicine to treat cutaneous eruptions from leishmaniasis, an infectious disease caused by protozoan parasites of the *Leishmania genus* [283]. Although urechitol A (**101**) itself exhibited no biological activity, its unique tetracyclic structure prompted some scientists to investigate its synthesis [284,285]. Until 2016, no knowledge existed about the accumulation dynamics of urechitol A (**101**) in wild plants of *P. andrieuxii*. However, results described by Peña-Rodríguez et al. indicated that the content of urechitol A (**101**) in root tissue was clearly related to plant development [286]. 

Several genetic transformation studies were conducted to gain insight into the production of this novel tri-*nor*-sesquiterpenoid, urechitol A (**101**). The *Agrobacterium rhizogenes* strain ATCC 15834 was used to infect leaf and hypocotyl explants of *P. andrieuxii* to generate 14 transformed plant lines with increased production of urechitol A. These new transgenic lines are promising tools to further the study and knowledge of the biosynthesis of terpenoids in *P. andrieuxii*, especially regarding the biosynthetic origin of the miscellaneous sesquiterpene urechitols [287].

## 7. Biosynthesis of 11,12,13-Tri-*nor*-Sesquiterpenes

### 7.1. Biosynthesis of 11,12,13-Tri-nor-Germacranes and Tri-nor-Elemanes

The 11,12,13-tri-*nor*-sesquiterpenes are irregular sesquiterpenoids which have lost the C_3_ unit of dimethylcarbinol at C-7 of the sesquiterpene skeleton. The irregular C-backbone originates from the oxidative removal of a C_3_ side chain from the C_15_ sesquiterpene, which arises from farnesyl diphosphate (FDP). Generally, in all families of sesquiterpenes, to generate the C_12_-framework, an oxidative cleavage of the C_3_ substituent with simultaneous introduction of a double bond has to occur [288]. However, some small variations to this general mechanism can be observed on different substrates or skeletons.

**Figure 10:**
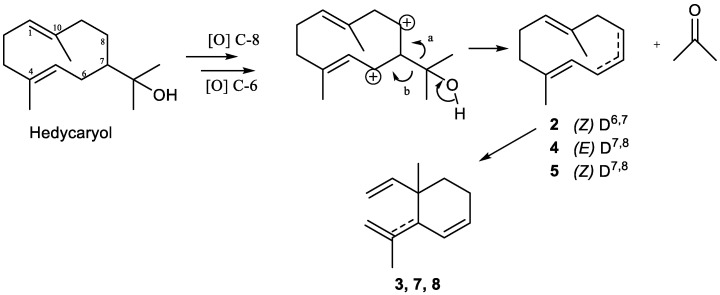
Proposed mechanism for biosynthesis of tri-*nor*-germacranes 2, 4 and 5, and tri-*nor*-elemanes 3, 7 and 8.

Tri-*nor*-germacranes have the same skeleton as germacranes, except for the oxidative lack of the isopropyl group, via enzymatic oxidation at C-8 or C-6, featuring a 12 carbon skeleton instead of a normal 15 carbon sesquiterpene skeleton (Figure 10) [8,65].

Thus, biosynthetically, pregeijerene (**2**) and isomers of pregeijerene B (**4**, **5**) can be considered derivatives of hedycaryol, which arise from FDP, via enzymatic oxidation at C-8 and C-6 [65], followed by an oxidative dealkylation of the dimethylcarbinol group generating an endocyclic double bond. This reaction strongly resembles the key step of the oxidative dealkylation of (+)-marmesin to psoralene [289] and, hence, might also be catalyzed by a cytochrome P450 [64]. Subsequently, tri-*nor*-germacranes can be isomerized to yield geijerene derivatives **3**, **7** and **8** by Cope rearrangement [27,89].

### 7.2. Biosynthesis of 11,12,13-Tri-nor-Eudesmanes

Eudesmanes are biosyntheszed by means of mevalonate pathways and involve the cyclization of farnesyl diphosphate (FDP) to give germacryl cation which yield the eudesmyl cation via transannular cyclization [5]. However, the 11,12,13-tri-*nor*-eudesmanes have generally been considered degraded sesquiterpenes where the irregular skeleton originates from oxidative removal of the C_3_ side chain. Recent studies have shown that, in some cases, the loss of the C_3_ unit was catalyzed by a special enzyme [144,290].

**Figure 11:**
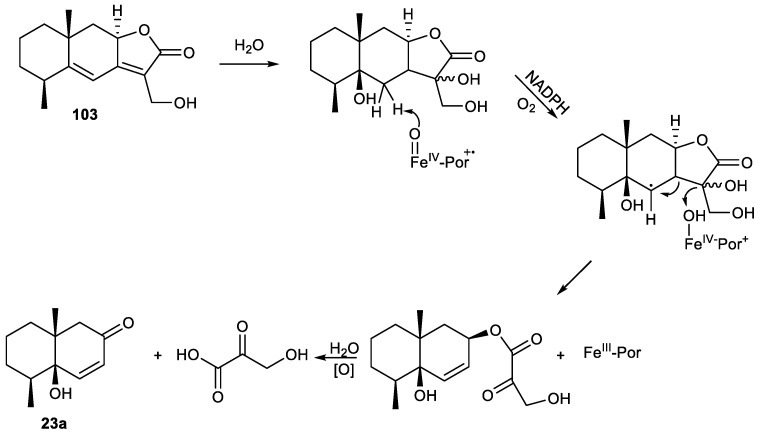
Proposed biosynthetic pathway to tri-*nor*-sesquiterpene by a special enzyme (adapted from Huang et al. 2010 [144]).

Hence, two sesquiterpenes were isolated from *Pulicaria insignis*, the C_12_ trinorsesquiterpene **23a** and sesquiterpene **103**, considered the precursor of **23a**, whose biosynthetic pathway is shown in Figure 11. Based on the work of Stanjek et al. [289], the loss of C_3_ units was considered to be mediated by a special enzyme [144].

Biosynthetic studies of 11,12,13-tri-*nor*-eudesmanes conducted in the 2000s have focused principally on the skeleton of geosmine, compound **28** probably being the most representative and important of the interesting family of tri-*nor*-sesquiterpenes. Geosmine (**28**) is produced by many bacteria, including actinomycetes, myxobacteria and cyanobacteria, as well as a number of eukaryotic organisms, such as fungi, liverworts, insects and plants [119,158,291,292]. This compound is responsible for the characteristic smell of moist soil or freshly plowed earth, and it is an important off-flavor contaminant of drinking water [293,294]. 

**Figure 12:**
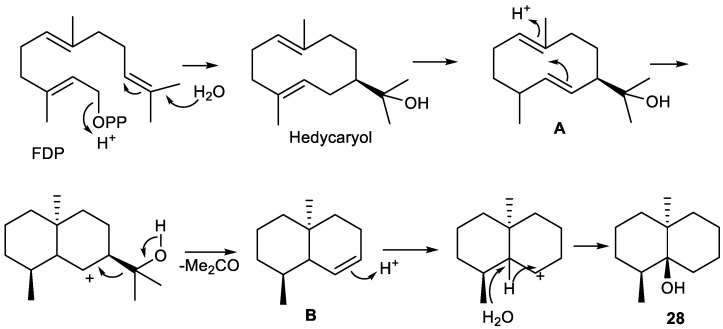
Biosynthesis of geosmin in *M. xanthus* and *S. aurantiaca* (adapted from Dickschat et al. 2005 [167]).

The biosynthetic pathway of this interesting compound remained unresolved for several decades and has triggered some controversy in the literature [120]. Despite being approached by various research groups, only recently have key experiments provided information on the mechanical details [120]. Initially, studies of the incorporation of deuterated precursors into geosmin (**28**) suggested that this bicyclic C_12_ metabolite might be a degraded sesquiterpene [64,295]. An explicit biosynthetic pathway in myxobacteria to geosmin (**28**) was proposed from feeding experiments with deuterium-labeled precursors [167]. The biosynthetic pathway to **28** was clarified by feeding small amounts of labeled leucine, dimethyl acrylate (DMAA) and mevalonic acid (MVA) to *Myxococcus xanthus* and *Stigmatella aurantiaca* that had been cultivated on agar plates. After feeding deuterated [^2^H_10_] leucine, Dickschat et al. [167] proposed a biosynthetic pathway to **28** with intermediate **A** similar in its early steps to the biosynthetic scheme postulated by Pollak and Berger [296] (Figure 12). 

The data obtained by Dickschat’s group were consistent with the proposed biosynthesis, but did not prove the intermediacy of A in the formation of **28**. The subsequent steps, namely cyclization to the bicyclic system, loss of acetone and the proton-mediated addition of water in combination with a 1,2-hydride shift, were consistent with the fragmentation pattern observed after feeding of the precursors [167]. 

The pathway proceeds from farnesyl diphosphate (FDP), which is cyclized to hedycaryol and further isomerized to (1(10)*E*,5*E*)-germacradien-11-ol (**A**). Protonation initiates the formation of the bicyclic carbon skeleton to give the C_12_ intermediate 8,10-dimethyl-1-octalin (**B**) that arises by cleavage of acetone.

Interestingly, the biosynthetic pathway to **28** was different from that previously described for the liverwort *Fossombronia pusilla* (sesquiterpenes formed via the mevalonate (MVA) pathway only) and *Streptomyces* sp. (sesquiterpenes can arise through the deoxyxylulose (DOX) phosphate pathway, as well as the mevalonate pathway, depending on the growth phase) [64]. 

**Figure 13:**
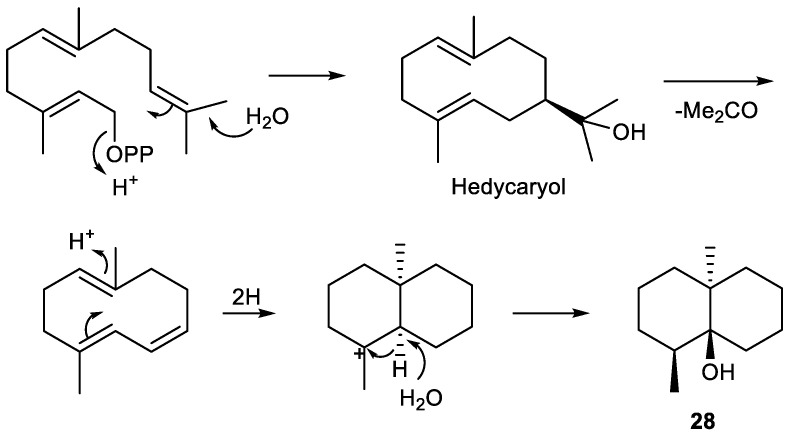
Biosynthesis of geosmin (**28**) in the liverwort *Fossombronia pusilla*.

Figure 13 represents the biosynthetic pathways to **28** in the liverwort *F. pusilla* in which the last step is characterized by a hydrogen shift of the same hydrogen, but into the left ring of **28**. The results of the feeding experiment with *F. pusilla*, employing deuterated mevalonic acid (MVA), clearly indicated the hydrogen shift into the left ring of **28**, giving strong evidence for the pathway outlined in Figure 13. The same mechanism has been suggested for *Streptomyces* sp. JP95 [64]. However, it was not possible to confirm the pathway operating in *Streptomyces* sp. or its possible dependence on the MVA or DOX pathways. Obviously, two independent pathways to **28** were proposed in nature [64,167].

**Figure 14:**
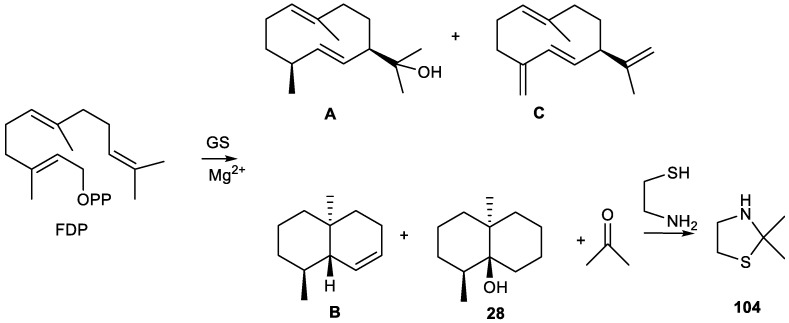
Cyclization/fragmentation of FDP to Geosmin by geosmin synthase (adapted from Jiang and Cane 2008 [298]).

The first characterized geosmin synthase was isolated from *Streptomyces coelicolor* A3(2) [290,292,297]. Expression in *Escherichia coli* of the SCO6073 and SC9B1.20 genes gave a 726 amino acid protein making up two catalytically active domains. The N-terminal domain converted FDP into a 85:15 mixture of (4*S*,7*R*)-germacra-1(10)*E*, 5*E*-diene-11-ol (**A**) and the sesquiterpene hydrocarbon (−)-(7*S*)-germacrene D (**C**), whereas the C-terminal domain, previously thought to be catalytically silent, catalyzed the Mg^2+^-dependent conversion of germacradienol (**A**) via the trinoreudesmane (**B**) to yield geosmin (**28**) (Figure 14) [119]. The mechanism of the fragmentation–rearrangement in the conversion of germacradienol (**A**) to geosmin (**28**) was studied by Jiang and Cane. These researchers reported evidence of the conversion of germacradienol (**A**) to geosmin (**28**) by *S. coelicolor* germacradienol/geosmin synthase resulting in the release of the three-carbon side chain as acetone and involving a 1,2-hydride shift of the bridgehead hydrogen exclusively into ring B of geosmin (**28**) [298]. To detect acetone generated in the formation of geosmin (**28**), the proposed fragmentation by-product acetone was trapped with cysteamine in an elegant experiment verifying the fate of the lost C_3_ unit. GC–MS analysis confirmed the formation of 2,2-dimethylthiazolidine (**104**) (Figure 14) [298]. 

Lastly, experiments conducted by Nawrath et al. [119] via synthesis of intermediate **B** and **10a** (Figure 3) unambiguously proved that both intermediates were formed by the geosmin synthase in streptomycetes, with **B** likely an intermediate and **10a** a shunt metabolite.

Later, the closely related geosmin synthases from *Streptomyces avermitilis* [299] and from cyanobacterium *Nostoc punctiforme* were isolated and shown to catalyze the same reaction as the *S. coelicolor* enzyme [120].

### 7.3. Biosynthesis of 11,12,13-Tri-nor-Eremophilanes

The biosynthesis of the eremophilane skeleton has been elucidated mainly by the application of stable isotopes and NMR spectroscopy. Synthesis follows the standard mevalonate pathway and involves cyclization of farnesyl diphosphate (FDP) to give the (*S*)-germacrene **A**, which is protonated in the C-6, C-7 double bond to give the bicyclic eudesmane cation. Successive 1,2 hydride shift and methyl migration, followed by loss of H_Si_ on C8, completes the generation of (+)-aristolechene [300]. 

**Figure 15:**
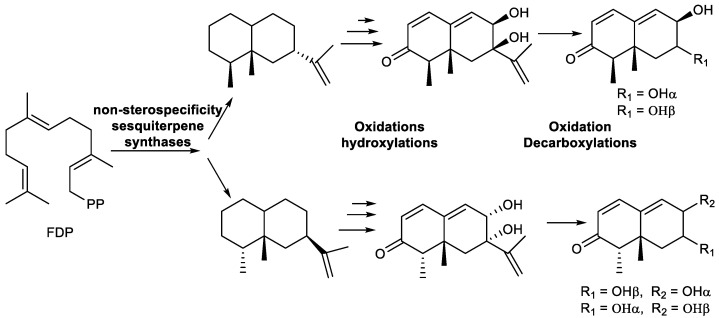
Proposed biosynthetic pathway to tri-*nor*-eremophilanes (adapted from Liu et al. 2016 [190]).

Formation of the tri-*nor*-eremophilanes is not known, but it has been proposed that the elimination of the isopropenyl group to give tri-*nor*-eremophilanes might occur via oxidation and subsequent decarboxylation (Figure 15) [190].

**Figure 16:**
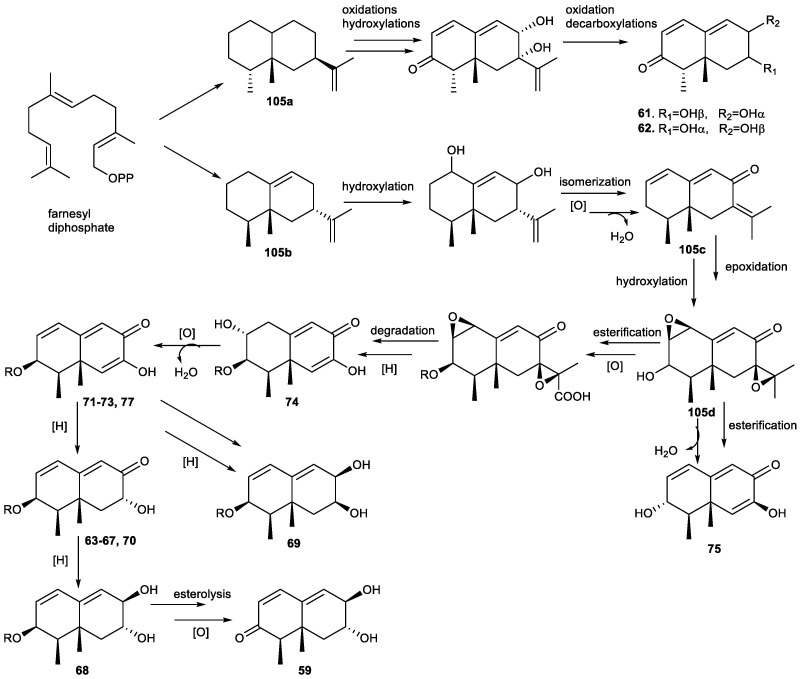
Hypothetical biosynthetic pathways of tri-*nor*-eremophilanes (adapted from Lin et al. 2021 [214]).

Different authors [190,214,218] have proposed that the tri-*nor*-eremophilanes (**59**, **63**–**77**, etc.) could originate from different precursors **105a**, **105b** and **105c**, which, after different types of tailoring reactions, including hydroxylation, oxidation, isomerization, epoxidation, esterification and degradation, might produce diverse structures (Figure 16) [190,214].

### 7.4. Biosynthesis of 11,12,13-Tri-nor-Guaianes

Natural tri-*nor*-guaianes are irregular metabolites that have been isolated from terrestrial, as well as marine sources [301]. Two of their most representative members are (−)-clavukerin A (**79**) and clavukerin C (**84**) (Figure 8), unstable dienes isolated from the Okinawan soft coral *Clavularia koellikeri* (stolonifer) by Kobayashi et al. in 1983 [219] and 1984 [231,241]. 

The terpenoid origin of tri-*nor*-guaianes was confirmed by the biosynthesis of 3,10-dihydro-1,4-dimethylazulene (**95**) [272] and by Dai et al. [302] in the biosynthesis of **79** in a *Heteroxenia* sp.

**Figure 17:**
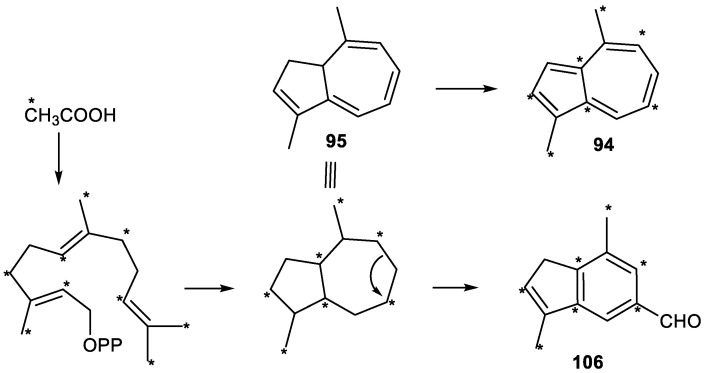
Incorporation of ^13^C from [2-^13^C]-labeled acetate into compounds **94**, **95** and **106** (adapted from Takeda and Katoh 1983b [272]).

The terpenoid origin of tri-*nor*-guaianes, and specifically of 3,10-dihydro-1,4-dimethylazulene (**95**), was confirmed by Takeda and Katoh in 1983 [272] via biosynthetic studies employing ^13^C-labeled acetate and different ^13^C NMR techniques of cultured cells of *Calypogeia granulate* (liverwort) [272]. The biosynthetic route leading to 3,7-dimethylindene-5-carboxaldehyde (**106**) was also clarified by ^13^C-labeling studies. The indene derivative is a trinorsesquiterpene which has undergone a skeletal rearrangement, as shown in Figure 17.

Furthermore, from a soft coral specie of genus *Heteroxenia*, de novo synthesis of the terpene clavukerin A (**79**) from sodium [1-^14^C] acetate and from D,L-[2-^14^C] mevalolactone was detected. The labeled acetate was incorporated with the expected selectivity, but degradation of the labeled mevalonate samples suggested some scrambling of the label, presumably via acetate incorporation of degraded mevalonate [302].

**Figure 18:**
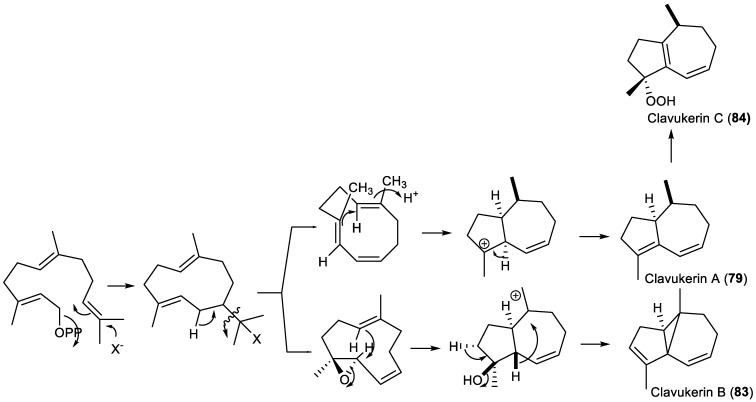
Hypothetical biogenetic pathway to clavukerins (adapted from Kobayashi et al. 1984b [241]).

The FA hypothetical biogenetic pathway to clavukerins A (**79**), B (**83**) and C (**84**) was proposed by Kobayashi et al. [241]. Their formation is presumably closely related to guaiane biosynthesis with the loss of the isopropyl side chain at an unknown stage along the biosynthetic pathway (Figure 18). A similar biosynthetic pathway has been proposed for the tri-*nor*-guaiane, 4β,10α-dimethyl-1β,5α-bicyclo [3,5,0] dec-6-en-4α,10β-diol (**89**) [264].

**Figure 19:**
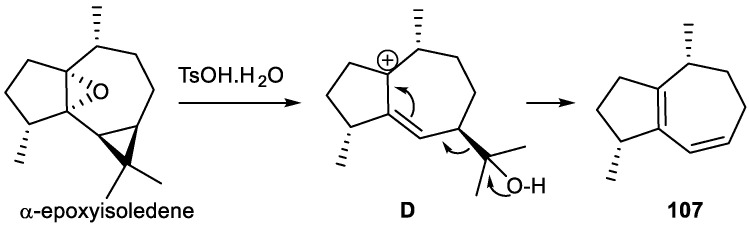
Treatment of α-epoxyisoledene with TsOH.H_2_O in acetone yielding tri-*nor*-guiadiene.

As previously indicated to generate the C_12_-framework, an oxidative cleavage [288] of the C_3_ substituent with simultaneous introduction of a double bond must occur. This oxidative degradation of isopropyl or the isopropenyl side chain has been confirmed by synthetic methods [231,303]. De Groot et al. have reported the formation of tri-*nor*-guaiane (**107**) at 20% yield when α-epoxyisoledene was treated with TsOH.H_2_O in acetone at room temperature. Its formation was explained by acetone elimination from allylic carbocation D (Figure 19). A bioinspired approach to the tri-*nor*-guaianes, clavukerin A (**79**), by degradation of the C-7 side chain of related guaia-11-enes, has also been described [231].

## 8. Conclusions

This review describes a comprehensive account of all reported sesquiterpenes, which have lost the C-3 unit of isopropenyl at C-7 position of the sesquiterpene skeleton. A total of one hundred and thirty-one 11,12,13-tri-*nor*-sesquiterpenes have been isolated from a vast number of different organisms. 

Based on their skeletons, five tri-*nor*-germacranes and four tri-*nor*-elemanes have been isolated. They displayed a wide range of antimicrobial bioactivity. Tri-*nor*-germacranes have been identified as components of essential oils (EO), and some, such as compounds **1**, **2**, **4**–**6**, have been extracted from the essential oils of different plants. However, geijerene (**3**) and isomers **7** and **8** are considered thermal artefacts of pregeijerene (**2**), which can be thermally isomerized to yield geijerene (**3**) by Cope rearrangement and chemical transformations (Figure 2). 

The bigger group of tri-*nor*-sesquiterpenes correspond to those with an underlying eudesmane skeleton (sixty tri-*nor*-eudesmanes have been reported, twenty of which are derived from geosmin (**28**); see Figure 3, Figure 4 and Figure 5). Most of tri-*nor*-eudesmanes have been isolated from different plant families, although some of them have been isolated from other organisms, such as Red Sea soft coral. All of them displayed a wide range of biological activities. 

Geosmin was first isolated from the actinomycete *Streptomyces griseus* by Gerber and Lechevalier [158], and it has also been isolated from a variety of higher plants, such as liverwort and sugar beet [166], and from mosses, protozoans and insects [64,167]. Environmentally relevant concentrations of geosmin (**28**) affect the development, oxidative stress, apoptosis and endocrine disruption of embryo–larval zebrafish [169]. Some of their derivatives, such as dehydrogeosmine (**29**), have been reported as ingredients in pharmaceuticals, including tetrahydrocannabinol and cannabidiol for the treatment of chronic pain and opioid addiction [83].

On the other hand, thirty-eight tri-*nor*-eremophilenes have been isolated—most of them from terrestrial plants—but in 1988, the study of the secondary metabolism of the marine deuteromycete *Dendryphiella salina* led to the isolation and characterization of the first tri-*nor*-eremophilane, dendryphiellin A (**63**), esterified by a branched C_9_ acid, a class of metabolite for which there is no precedent in fungi of marine origin. Subsequently, approximately 12 new derivatives of dendryphiellin A (**63**–**74**, **77**) were isolated from different organisms. Although an important range of biological activity has been described, it is important to emphasize the biological activity shown by compounds **54** and **55**, which were proved as potential candidates for the development of therapeutically relevant agents to prevent neurodegenerative diseases [206]. 

Finally, eighteen tri-*nor*-sesquiterpenes with guaiane skeleton and six with skeletons not classified in the previous groups complete the set of tri-*nor*-sesquiterpenes isolated from nature.

About biosynthesis, in general, the irregular C-backbone originates from the oxidative removal of a C_3_ side chain from the C_15_ sesquiterpene, which arises from farnesyl diphosphate (FDP). However, recent studies have shown that, in some cases, such as geosmin (**28**), the loss of the C_3_ unit was catalyzed by a special enzyme. These authors have demonstrated that geosmin was biosynthesized by geosmin synthase, an enzyme characterized from *Streptomyces avermitilis* [299], and from cyanobacterium *Nostoc punctiforme*, which catalyzes the same reaction as the *S. coelicolor* enzyme [144,290]. These studies and conclusions about the reported geosmine synthase open new and interesting ways to study the biosynthetic pathways of other trinorsequiterpenes.

## Data Availability

Not applicable.
